# Interaction-induced edge states in anisotropic non-Fermi liquids

**DOI:** 10.1038/s41598-017-03823-5

**Published:** 2017-06-14

**Authors:** I. V. Yurkevich

**Affiliations:** 1 Nonlinearity and Complexity Research Group, School of Engineering & Applied Science, Aston University, Birmingham, B4 7ET United Kingdom; 2 Institute of Fundamental and Frontier Sciences, University of Electronic Science and Technology of China, Chengdu, 610054 People’s Republic of China

## Abstract

We devise an approach to calculation of scaling dimensions of generic operators describing scattering within multi-channel Luttinger liquid. The local impurity scattering in arbitrary configuration of conducting and insulating channels is investigated and the problem is reduced to a single algebraic matrix equation. The application to a semi-infinite array of chains described by Luttinger liquid models demonstrates that for a weak inter-chain hybridisation and intra-channel electron-electron attraction the edge wire is robust against disorder whereas bulk wires, on contrary, become insulating in some region of inter-chain interaction parameters. This result proves that the edge states may exist in disordered anisotropic strongly correlated systems *without* time-reversal symmetry breaking or spin-orbit interaction and provide quantized low-temperature transport.

## Introduction

The recent advances in study of strongly correlated systems has led to wider search for exotic non-Fermi-liquid states in condensed matter systems. In particular, the quest for edge states protected against disorder has started. These states are protected by a symmetry forbidding perturbations that are potentially dangerous for the phase stability. The perturbations presenting a threat to the stability may also be forbidden by a conservation law. Another option is a renormalisation of the dangerous perturbations by the particle correlations such that the scattering processes become suppressed at low temperatures and leading to a ‘protected’ zero-temperature state. One of the promising models providing rich non-Fermi-liquid physics is an anisotropic system consisting of array of coupled one-dimensional wires. This model was used to for construction of integer^1^ and fractional quantum Hall states^2^. Sliding phases in classical *XY* models^3^, smectic metals^4^ and many other exotic states are all described by the sliding Luttinger liquid (sLL) model^5^. Infinite arrays of wires and few-channel liquids have been investigated for decades. Their stability against disorder is another wide area of research. A single impurity embedded into a Luttinger liquid^6, 7^, and a continuous disorder in strongly correlated electron liquids^8–10^, are known to be essential for a single channel problem. The renormalisation group (RG) analysis shows that transport of repulsive electrons is completely blocked by a single impurity at zero temperature^6, 7^. The role of disorder (local impurity) embedded into a Luttinger liquid (LL) coupled to a quasi-one-dimensional quadratic bath was explored in refs 11–13. Multi-channel strongly correlated systems with disorder have not been studied yet. The objective of this paper is to study a local impurity coupled to a multi-channel Luttinger liquid.

We will develop generic approach allowing treatment of various realisations of multi-channel Luttinger liquids where channels are either labeled by quantum numbers or spatially arranged to form an array of wires. In particular, we investigate effect of a single impurity onto a finite array of weakly coupled Luttinger liquids (sliding Luttinger liquid). Our results also describe an array of wires with low density of impurities at moderate temperatures when thermal length is smaller than the mean distance between impurities and, therefore, each impurity renormalises individually. We also assume that hybridisation, *t*_⊥_, between channels (wires) is small enough to ensure that the temperature, *T*_⊥_ ~ *D*(*t*_⊥_/*D*)^*α*^ (where *D* is the bandwidth and the exponent *α* = (2 − Δ_⊥_)^−1^ is defined through the scaling dimension, Δ_⊥_, of the most dangerous hybridization term) is low enough and cannot be reached. Under these assumptions we may safely use model of interacting but not hybridised wires (channels) with disorder modelled by the individual impurities. And under these assumptions we will demonstrate that in some region of parameters (intra- and inter-wire interaction strengths) we must observe the edge states in 2D array of correlated wires without both magnetic field (preserved time-reversal symmetry) and spin-orbit scattering. The inter-wire interaction may enhance impurity scattering in the bulk wires (making them insulating) and suppress impurity scattering in the edge wires. This behaviour is similar to the one observed in quantum Hall systems or topological insulators.

It is known that RG flow for a single channel problem depends on single parameter (so-called Luttinger parameter) K while excitations velocity does not play any role in the renormalisation^6, 7^. Our general construction generalises this result: *N*-channel LL is described by *N* velocities and a real symmetric *N* × *N* matrix which we call Luttinger $\hat{K}$-matrix responsible for the impurity strength renormalisation. This matrix must be found from the algebraic matrix equation

1 $$\hat{K}\,{\hat{V}}_{+}\,\hat{K}={\hat{V}}_{-}\,,$$

where the matrices ${\hat{V}}_{+}$ and ${\hat{V}}_{-}$ describe density-density and current-current (both intra- and inter-channel) interactions between particles. It is this ‘Luttinger’ $\hat{K}$-matrix that defines the scaling dimensions of all possible scattering terms in all possible phases. A phase is a particular set of *N* channels each of which is either continuous (conducting) or snipped (insulating) at the position of the impurity. To establish whether a phase is stable one has to check relevance of all thought perturbations. The most general perturbation corresponds to the process when *n*_*i*_ particles are backscattered (tunneled) in conducting (insulating) *i*-th channel. The scaling dimension of the operator describing such a process is given by:

2 $${\rm{\Delta }}[{\bf{n}}]={{\bf{n}}}^{{\rm{T}}}\,\hat{{\rm{\Delta }}}\,{\bf{n}}\,,\,{\hat{{\rm{\Delta }}}}^{-1}={{\mathscr{P}}}_{{\rm{c}}}\,{\hat{K}}^{-1}{{\mathscr{P}}}_{{\rm{c}}}+{{\mathscr{P}}}_{{\rm{i}}}\,\hat{K}{{\mathscr{P}}}_{{\rm{i}}}\,,$$

Here ${{\mathscr{P}}}_{{c}/{i}}$ are the projectors onto conducting/insulating channels subspaces and **n** ^**T**^ = (*n*_1_, …, *n*_N_) is the integer-valued vector describing multiplicities of intra-channel scatterings. The complete and refined derivation of our main Eqs (1) and (2) can be found in the Appendixes B and E of the Supplementary Information (SI).

Our main result will be the application of this generic approach to a finite 2D array (strip) and the proof of the existence of the edge states robust against disorder on the background of insulating bulk. As it will be seen later, such a situation is realised for a weak intra-channel attraction between fermions or rather strong repulsion between bosons. The situation is similar to what is observed in quantum Hall systems or topological insulators. The main difference is that we describe situation when quantized edge currents require neither time reversal symmetry breaking nor spin-orbit interaction with non-trivial topology of single-particle spectrum. The concept of interaction-protected conducting phases can be also found in the paper^14^ where the authors essentially revisited analysis performed in earlier papers^2, 5^, and modernised it in application to the topological insulators (by mere exclusion of intra-channel single-particle backscattering forbidden by the time-reversal symmetry). All those papers considered either infinite arrays^2, 5^, or effectively arrays on a cylinder (periodic boundary conditions^14^) thus missing the opportunity of the boundary effects. To the best of our knowledge, the edge states in the sliding Luttinger liquid model were not predicted before.

## The model

The Lagrangian describing a multichannel Luttinger liquid is built on two vector fields, ***θ*** = (*θ*_1_, …, *θ*_*N*_) and ***ϕ*** = (*ϕ*_1_, …, *ϕ*_*N*_), parameterising excitation densities, *ρ*_*i*_ = ∂_*x*_ *θ*_*i*_/2*π*, and currents, *j*_*i*_ = ∂_*x*_ *ϕ*_*i*_/2*π*, in each channel *i* (1 ≤*i* ≤ *N*))^1–5^. The Lagrangian, ${ {\mathcal L} }_{0}$,

3 $${ {\mathcal L} }_{0}=\frac{1}{2}{{\boldsymbol{\Psi }}}^{{\rm{T}}}\,{\hat{G}}_{0}^{-1}\,{\boldsymbol{\Psi }}\,,\,{\hat{G}}_{0}^{-1}=\frac{1}{4\pi }\,[{\hat{\tau }}_{1}\,{\partial }_{t}+\hat{V}\,{\partial }_{x}]\,{\partial }_{x},\,{{\boldsymbol{\Psi }}}^{{\rm{T}}}=({{\boldsymbol{\theta }}}^{{\rm{T}}},{{\boldsymbol{\varphi }}}^{{\rm{T}}}\mathrm{).}$$

includes block-diagonal matrix $\hat{V}={\rm{diag}}[{\hat{V}}_{+}\,,{\hat{V}}_{-}]$ with each block describing density-density, ${\hat{V}}_{+}$, and current-current, ${\hat{V}}_{-}$, interactions (see SI Appendix A); ${\hat{\tau }}_{1}$ is the Pauli matrix.

Perturbing translation invariant system by a local impurity, one may find that the system is unstable in the sense that the scaling dimension of this perturbation is smaller than one (which is the physical dimension of a local term in 1 + 1 dimensional system). This means that a perturbation theory would be divergent and allegedly translation invariant configuration was a bad initial guess. In terms of transport, this fact suggests that some (all or few) of the channels (wires) become insulating and not ideally conducting as it is assumed in a translation invariant configuration. To test stability of such an inhomogeneous configuration where some channels are conducting while others are insulating, we have to introduce two subspaces of conducting, **C**, and insulating, **I**, channels. Since we have no *á priori* knowledge which configuration will be stable (and, therefore, may be called a phase) against all legitimate perturbations, we must devise a generic approach allowing treatment of all possible configurations.

The translation invariant channels, *i* ⊂ **C**, are described by continuous *θ*_*i*_ - and *ϕ*_*i*_ -fields. The insulating channels, *i* ⊂ **I**, are cut at the origin and, therefore, described by the fields obeying the boundary conditions imposing zero current^15^, ${j}_{i}(x,t)=-{\partial }_{t}{\theta }_{i}(x,t\mathrm{)/2}\pi$):

4 $$\{\begin{array}{ll}{\theta }_{i}(-\mathrm{0)}={\theta }_{i}(+\mathrm{0),}\,{\varphi }_{i}(-\mathrm{0)}={\varphi }_{i}(+\mathrm{0)}, & i\subset C;\\ {\theta }_{i}(-\mathrm{0)}={\theta }_{i}(+\mathrm{0)}=\mathrm{0,}\,{\partial }_{x}{\varphi }_{i}(-\mathrm{0)}={\partial }_{x}{\varphi }_{i}(+\mathrm{0),} & i\subset I.\end{array}$$

Instead of using this boundary conditions, we find it more convenient to do the following trick: adding to the translation invariant Lagrangian, ${ {\mathcal L} }_{0}$, a quadratic form with an auxiliary diagonal kernel matrix $\hat{\zeta }={\rm{diag}}({\zeta }_{1},\,\mathrm{...},\,{\zeta }_{N})$. This matrix will not affect the channel *i* if the corresponding parameter is set to zero *ξ*_*i*_ → 0, whereas it will suppress current (like an infinite barrier) if *ξ*_*i*_ → ∞. One can derive all necessary correlations for an arbitrary $\hat{\xi }$-matrix because the auxiliary Lagrangian is still quadratic,

5 $${ {\mathcal L} }_{\xi }={ {\mathcal L} }_{0}-\frac{1}{2}{{\boldsymbol{\theta }}}^{{\rm{T}}}\,\hat{\zeta }{\boldsymbol{\theta }},$$

and remove it later from the final result using the limiting procedure

6 $${\zeta }_{i}\,\to \,\{\begin{array}{c}0\,,\,i\subset {\bf{C}}\,,\\ \infty \,,\,i\subset {\bf{I}}\,\mathrm{.}\end{array}\,\mathrm{.}$$

The correlations found from the auxiliary Lagrangian ${{ {\mathcal L} }}_{\xi }$ become true correlations of an inhomogeneous configuration after this limit is performed. The trick allows dealing with all configurations on the same footing. In what follows, we will refer to this limiting procedure simply as *ξ*-limit (the name will become clear later when *ζ* will be rescaled and renamed to *ξ*).

## Results

In the regime of a weak inter-wire hybridisation one can neglect impurity scattering resulting in particles hopping between the wires in the course of their scattering. Limiting ourselves to the simplest model of the nearest neighbours inter-channel interaction, both density-density, ${\hat{V}}_{+}$, and current-current, ${\hat{V}}_{-}$, interaction matrices become symmetric three-diagonal matrices. All such matrices commute with each other and, therefore, we can explicitly solve the Eq. (1) defining the Luttinger mutrix:

7 $$\hat{K}=\sqrt{\frac{{\hat{V}}_{-}}{{\hat{V}}_{+}}}\,\mathrm{.}$$

The most dangerous intra-wire process is the single-particle backscattering. When it happens in the *i*-th wire, the scaling dimension of such a process is given by (see Methods)

8 $${{\rm{\Delta }}}_{i}={K}_{ii}=2K{\int }_{0}^{\pi }\,\frac{{\rm{d}}q}{\pi }{\kappa }_{q}\,{\sin }^{2}\,qi,\quad {\kappa }_{q}=\sqrt{\frac{1+{\alpha }_{-}\,\cos \,q}{1+{\alpha }_{+}\,\cos \,q}}.$$

Here *K* is the standard Luttinger parameter defined under the absence of inter-wire interactions: *K* < 1 describes single-wire fermions with repulsion and *K* > 1 corresponds to either attractive fermions or repulsive bosons^16^. The parameters *α*_±_ are dimensionless strengths of interaction between the adjacent wires. They are proportional to the off-diagonal elements of ${\hat{V}}_{\pm }$-interaction matrices in the Eq. (3). The explicit expressions for the inter-wire density-density, (*α*_±_), and current-current, (*α*_−_), interactions are not universal. Their dependence on bare values of interactions and the mean particle density differ for bosons and fermions and, therefore, we will not dwell on these details. The only important fact is that the positive definiteness of interactions implies that |*α*_±_| < 1 and *α*_±_ is positive (negative) for repulsion (attraction) while the sign of *α*_−_ can be arbitrary reflecting competition in the density-density and current-current sectors.

The backscattering in the bulk (*i* ≫ 1) and at the edge (*i* = 1) have different scaling dimensions, Δ_bulk_

9 $${{\rm{\Delta }}}_{{\rm{b}}{\rm{u}}{\rm{l}}{\rm{k}}}=\frac{K}{{K}_{{\rm{b}}{\rm{u}}{\rm{l}}{\rm{k}}}},\,{{\rm{\Delta }}}_{{\rm{e}}{\rm{d}}{\rm{g}}{\rm{e}}}=\frac{K}{{K}_{{\rm{e}}{\rm{d}}{\rm{g}}{\rm{e}}}},\quad {\rm{w}}{\rm{h}}{\rm{e}}{\rm{r}}{\rm{e}}\,{K}_{{\rm{b}}{\rm{u}}{\rm{l}}{\rm{k}}}^{-1}={\int }_{0}^{\pi }\,\frac{{\rm{d}}q}{\pi }\,{\kappa }_{q},\,{K}_{{\rm{e}}{\rm{d}}{\rm{g}}{\rm{e}}}^{-1}=2{\int }_{0}^{\pi }\,\frac{{\rm{d}}q}{\pi }\,{\kappa }_{q}\,{\sin }^{2}\,q.$$

which are found from the general expression Eq. (8). As one can see from Eq. (9), in the regions of parameters

10 $${K}_{{\rm{e}}dge} < K < {K}_{{\rm{b}}ulk}\,,$$

the impurity scattering in the bulk is RG-relevant and drives the system into the bulk insulator phase whereas backscattering at the edge is irrelevant and the edge wire is perfectly conducting. The system then is similar to a quantum Hall system or a topological insulator: the bulk is insulating (low-temperature transport is blocked by backscattering from individual impurities in each bulk wire) whereas edge wires are robust against backscattering generated by an impurity sitting at the edge.

**Figure 1:**
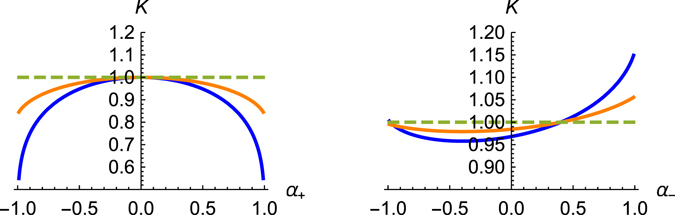
The critical scaling dimensions, *K*_bulk_ (blue) and *K*_edge_ (orange) dependence on (**a**) density-density inter-wire interaction strength, *α*_+_ without current-current inter-channels interactions, *α*_*−*_ = 0, and (**b**) current-current inter-wire interaction *α*_*−*_ for a given strength of inter-wire density-density interaction, *α*_+_ = 0.4.

Both critical values of the intra-wire Luttinger parameter, *K*_bulk_ and *K*_edge_, separating conducting and insulating phases of the bulk (*K* > *K*_bulk_ or *K* < *K*_bulk_), and the edge (*K* > *K*_bulk_ or *K* < *K*_bulk_), are plotted in the Fig. 1. The Fig. 1(a) presents the critical values, *K*_bulk_ and *K*_edge_, in the case of the absence of current-current interactions, *α*_−_ = 0. The critical values are function of density-density interaction strength *α*_+_, they both are smaller than one and are decreasing functions of interaction. The density-density interaction of a wire with its surrounding generates intra-wire attraction between electrons (BCS-type effect). The effective attraction drives the system towards conductor making impurity scattering less relevant (similar conclusion has been reached in ref. 14). This picture corresponds to the suppression of the critical *K*-values. It is obvious that the effect of surrounding is more pronounced in the bulk than at the edge leading to a weaker suppression of the edge critical value, *K*_bulk_ ≤ *K*_edge_ ≤ 1. Therefore, without current-current inter-wire interactions, both bulk and edge wires are either conducting for *K* > *K*_edge_ or driven towards insulating phases, *K* < *K*_bulk_. The intermediate regime, *K* < *K*_bulk_ < *K* < *K*_edge_, corresponds to the conducting bulk and insulating edge. This regime is unappealing since it is nothing more that just a negligible reduction of the number of conducting bulk wires.

The situation is completely different when current-current interactions win over density-density inter-wire interactions, *α*_−_ > *α*_+_. As one can see in the Fig. 1(b), strong inter-wire current-current interaction is equivalent to a suppression of the scaling dimensions or, in other words, increase of the critical values, *K*_bulk_ and *K*_edge_. The fact that the surrounding now leads to the induced additional intra-wire repulsion can be understood from the duality arguments based on the Eq. (7). It is seen that swapping density and currents, ${\hat{V}}_{+}\leftrightarrow {\hat{V}}_{-}$, leads to the replacement $\hat{K}\to {\hat{K}}^{-1}$. The current-current and density-density interactions play opposite roles and compete against each other. The effect of the surrounding onto the bulk and edge wires always favours edge states due to its relatively (with respect to the bulk) limited exposure to it. That is why the increase of the bulk and the edge critical values is more dramatic for the bulk one, *K*_edge_ < *K*_bulk_, for strong enough inter-wire current-current interaction, *α*_−_ > *α*_+_. Restricting ourselves to the region *α*_−_ > *α*_+_, we can observe that on the top of the anticipated phases, when all wires are either conducting or insulating, we can reach the intermediate regime *K*_edge_ < *K* *<* *K*_bulk_ predicted earlier, Eq. (10). In this regime, a local impurity at the edge becomes RG-irrelevant while the same impurity placed in the bulk wires is relevant. Hence the bulk wires are driven into the insulating regime whereas edge wire tends to become ideally conducting. This situation arises when we have weak intra-channel attraction between fermions (or strong repulsion between bosons) since this intermediate state is possible for *K* > 1 (see Fig. 1).

**Figure 2:**
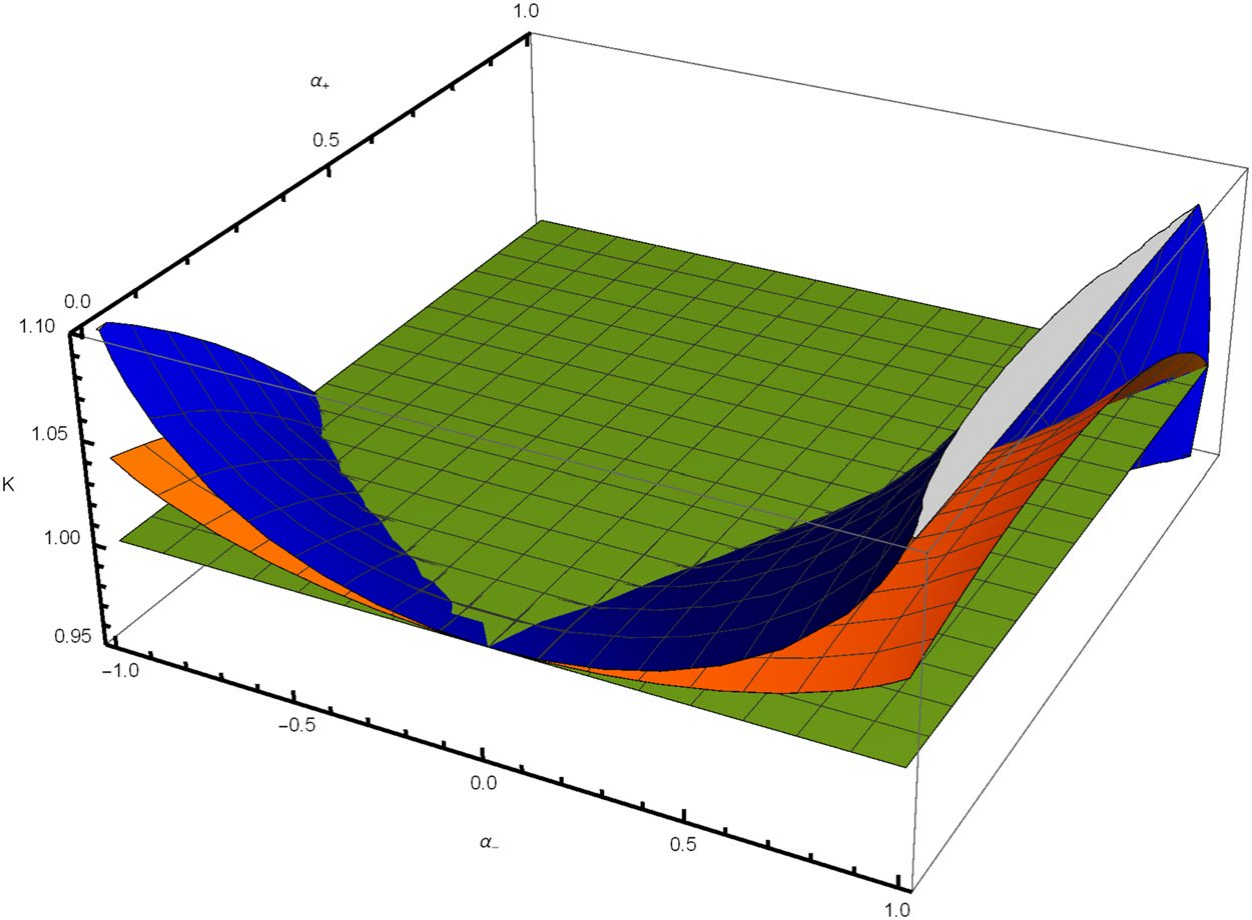
The critical scaling dimensions, *K*_bulk_ (blue) and *K*_edge_ (orange), as functions of inter-wire interaction parameters *α*_+_ and *α*_−_. The region below the blue and above the yellow surfaces corresponds to the phase with conducting edge states surrounded by insulating bulk wires. The green plane, *K* = 1, is for eye guidance only.

In the Fig. 2, one can see values of the critical scaling dimensions for the weak intra-channel attraction, *K* = 1.05. The plot presents the critical values *K*_edge_ (blue surface) and *K*_bulk_ (orange surface) as the functions of density-density and current-current inter-wire interaction strengths, *α*_+_ and *α*_+_. The green surface is the *K* = 1 plane. The region between the blue, *K*_bulk_, and the orange, *K*_edge_, surfaces corresponds to the region Eq. (10) of parameters *α*_+_ with conducting edge states and insulating bulk.

The only subtle point that needs attention is the necessity to test whether this intermediate state is a stable fixed point. The problem is that so far we have tested only the situation when the bulk is insulating (irrelevant of the edge state, the bulk cannot depend on a state of a single edge wire) while the edge state is translation invariant but perturbed by a weak back scatterer. It means that we have proved the stability of the conducting edge state against weak perturbation. But we know that there are so-called mixed states where both conducting and insulating phases are equally available and the actual state depends on the scattering strength. Such states correspond to the unstable RG fixed points. To classify the RG fixed point, one has to test the configuration where the edge wire is cut in two pieces that are inter-connected by a weak tunnel link (similar to the approach of refs 6 and 7). If the tunneling turns out to be a relevant perturbation, then and only then one may claim that the conducting phase of the edge state survives, the edge state is robust against any perturbation strength and the conducting edge is the true phase. Otherwise, the edge state would be in a mixed state (see ref. 17 for a detailed discussion). As it is shown in the Supplemental Information, the scaling dimension ${{\rm{\Delta }}}_{{\rm{e}}{\rm{d}}{\rm{g}}{\rm{e}}}^{{\rm{t}}{\rm{u}}{\rm{n}}}$ of the corresponding tunneling operator can be extracted from the generic result on dualities in multi-channel luttinger liquids (Eq. (F.12) of the Supplemental Information): ${{\rm{\Delta }}}_{{\rm{e}}{\rm{d}}{\rm{g}}{\rm{e}}}^{{\rm{t}}{\rm{u}}{\rm{n}}}={{\rm{\Delta }}}_{{\rm{e}}{\rm{d}}{\rm{g}}{\rm{e}}}^{-1}\mathrm{.}$ This duality means that only one of the edge state phases (conducting or insulating) can be stable, i.e. only one of the scaling dimensions can be smaller than one. This is the situation of a single stable fixed point when the conducting edge state is robust against both weak and strong scattering. Therefore, in the range of parameters defined by the inequality Eq. (10) the edge state is’protected’ by inter-wire interactions at low-temperatures.

## Methods

The auxiliary Lagrangian ${ {\mathcal L} }_{\xi }$ in the Eq. (5) involves the auxiliary Green function ${\hat{G}}_{\xi }$ that can be calculated using ***T***-matrix technique. The equation for retarded Green function in (*ω*,*x*)-representation is given by

11 $${\hat{G}}_{\xi }^{{\rm{R}}}(\omega ;x,x^{\prime} )={\hat{G}}_{0}^{{\rm{R}}}(\omega ;x-x^{\prime} )+{\hat{G}}_{0}^{{\rm{R}}}(\omega ;x)\,{{\rm{T}}}_{\xi }^{{\rm{R}}}(\omega ){\hat{G}}_{0}^{{\rm{R}}}(\omega ;-x^{\prime} ),$$

where $\hat{T}$-matrix depends on rescaled auxiliary matrix $\hat{\xi }\mathrm{=(2}\pi \,i/\omega )\,\hat{\zeta }$:

12 $${\hat{T}}_{\xi }=\frac{\omega }{2\pi i}(\begin{array}{cc}{\hat{R}}_{\xi } & 0\\ 0 & 0\end{array}),\,{\hat{R}}_{\xi }=\frac{1}{\hat{K}+{\hat{\xi }}^{-1}}.$$

The local Green function ($x,x^{\prime} \to \pm 0$) is found from the algebraic Eq. (11). The *ξ*-limit (which is not universal and dependent on the phase under consideration) should then be taken to obtain the true correlations of the fields.

The most general term describing a process of simultaneous backscattering of few particles in the conducting channels and accompanying it transfer (tunneling) of particles across the cut in the insulating channels is written in the terms of the field **Φ** = ***θ***_c_ + ***φ***_**i**_ as

13 $${ {\mathcal L} }_{{\rm{pert}}}=\sum _{{\bf{n}}}\,{ {\mathcal L} }_{{\bf{n}}},\quad { {\mathcal L} }_{{\bf{n}}}={v}_{{\bf{n}}}\,{e}^{i{{\bf{n}}}^{{\rm{T}}}{\boldsymbol{\Phi }}}+{\rm{c}}{\rm{.c}}.,$$

where we have defined integer-valued vector **n** ^T^ = (*n*_1_, *n*_2_, …, *n*_*N*_) and a new field ${\boldsymbol{\phi }}=[{\boldsymbol{\varphi }}(x=+0)-{\boldsymbol{\varphi }}(x=-0)]\mathrm{/2}$ describing the old *ϕ*-fields discontinuity around the scatterer (placed at the origin). There are two different processes that appear in the scattering term Eq. (13). First, it is backscattering in conducting channels where only ***θ***-fields from conducting channels, ${{\boldsymbol{\theta }}}_{{\rm{c}}}\equiv {{\mathscr{P}}}_{{\rm{c}}}{\boldsymbol{\theta }}$, define this process. Second, it is the tunneling in the insulating channels governed by the fields discontinuity ${{\boldsymbol{\phi }}}_{{\rm{i}}}\equiv {{\mathscr{P}}}_{{\rm{i}}}{\boldsymbol{\phi }}$. The Eq. (13) represents the most general perturbation that can be applied to an arbitrary configuration if we neglect (as it was discussed in the introduction) scattering accompanied by a hopping between the wires. We would like to stress here that we restrict ourselves to these perturbations only to keep the calculations simple. If one needs and account of a specific scattering process, the only thing which has to be done is the relabeling of *chiral* channels to make sure that the important scattering event occurs inside the same non-chiral channel. Such a procedure will corrupt the matrices ${\hat{V}}_{\pm }$ but otherwise the whole approach will be intact.

All the necessary for our purpose correlations are contained in the reduced Green function:

14 $$i{{\mathscr{G}}}_{\xi }=(\begin{array}{cc}\langle {{\boldsymbol{\theta }}}_{{\rm{c}}}\otimes {{\boldsymbol{\theta }}}_{{\rm{c}}}^{{\rm{T}}}\rangle  & \langle {{\boldsymbol{\theta }}}_{{\rm{c}}}\otimes {{\boldsymbol{\phi }}}_{{\rm{i}}}^{{\rm{T}}}\rangle \\ \langle {{\boldsymbol{\phi }}}_{{\rm{i}}}\otimes {{\boldsymbol{\theta }}}_{{\rm{c}}}^{{\rm{T}}}\rangle  & \langle {{\boldsymbol{\phi }}}_{{\rm{i}}}\otimes {{\boldsymbol{\phi }}}_{{\rm{i}}}^{{\rm{T}}}\rangle \end{array}),$$

that can be extracted from the local (*x*,*x′* ± 0) limit of the Eq. (11). Performing *ξ*-limit (see SI Appendix E), the scaling dimensions, Δ[**n**], of the exponentials in Eq. (13) can be written as the quadratic forms,

15 $${\rm{\dim }}[{ {\mathcal L} }_{{\bf{n}}}]={\rm{\Delta }}[{\bf{n}}]={{\bf{n}}}_{{\rm{c}}}^{{\rm{T}}}\,{[{\hat{{\mathscr{P}}}}_{{\rm{c}}}{\hat{K}}^{-1}{\hat{{\mathscr{P}}}}_{{\rm{c}}}]}^{-1}\,{{\bf{n}}}_{{\rm{c}}}+{{\bf{n}}}_{{\rm{i}}}^{{\rm{T}}}\,{[{\hat{{\mathscr{P}}}}_{{\rm{i}}}\hat{K}{\hat{{\mathscr{P}}}}_{{\rm{i}}}]}^{-1}\,{{\bf{n}}}_{{\rm{i}}},$$

where ${{\bf{n}}}_{{\rm{c}}}={{\mathscr{P}}}_{{\rm{c}}}{\bf{n}}$ and ${{\bf{n}}}_{{\rm{i}}}={{\mathscr{P}}}_{{\rm{i}}}{\bf{n}}$. The inversion of the projected matrices in Eq. (15) must be performed in their corresponding subspaces, conducting or insulating. Using properties of the projectors, the scaling dimensions can finally be written in the form presented earlier in the Introduction, Eq. (2).

To determine a scaling dimensions of a scattering operator, one has to solve the algebraic matrix Eq. (1) and plug the Luttinger $\hat{K}$-matrix into Eq. (2). This is be easily done in two rather simple limits that have been treated earlier otherwise^4, 5, 17^. But now we are interested in a *finite* array of spatially ordered wires. It turns out that this model can be treated analytically. The importance of this model lies in the fact that we may analyse boundary of multi-wire Luttinger liquid (surface or edge states depending of spatial arrangements). Below we will show that a solution of the Eq. (1) can be obtained for a finite strip in the nearest neighbours interaction model. This allows application of the scheme devised in this paper and, therefore, analysis of the edge wire stability against impurity scattering.

The nearest neighbours interaction model is described by a symmetric three-diagonal ${\hat{V}}_{\pm }$ interaction matrices:

16 $${V}_{\pm }^{ij}={v}_{\pm }\,{\delta }_{ij}+{v}_{\pm }^{^{\prime} }\,[{\delta }_{i,j+1}+{\delta }_{i,j-1}]\,\mathrm{.}$$

Here ${v}_{\pm }^{^{\prime} }$ describe inter-channel density-density and current-current interactions, while *v*_±_ encode intra-channel properties. Without inter-channel interaction we would have two noninteracting channels (wires) described by the velocity $v=\sqrt{{v}_{+}\,{v}_{-}}$ and the Luttinger parameter (describing intra-wire correlations of an isolated wire). Since all symmetric three-diagonal matrices are diagonalisable by the same orthogonal similarity transformation, the solution for the $\hat{K}$-matrix, Eq. (7), cane be written explicitly (see SI Appendix) and in the large-*N* limit this solution becomes:

17 $${K}_{ij}=2K{\int }_{0}^{\pi }\frac{{\rm{d}}q}{\pi }\,{\kappa }_{q}\,\sin \,qi\,\sin \,qj,\quad K=\sqrt{\frac{{v}_{-}}{{v}_{+}}},\quad {\kappa }_{q}=\sqrt{\frac{1+{\alpha }_{-}\,\cos \,q}{1+{\alpha }_{+}\,\cos \,q}},\quad {\alpha }_{\pm }=\frac{2{v}_{\pm }^{^{\prime} }}{{v}_{\pm }}.$$

The intra-wire correlations are defined by the standard Luttinger parameter *K*, while the rest describes the effect of the inter-channel interactions. The parameters *α*_±_ are dimensionless strengths of the density-density and current-current inter-channel interactions. The substitutions ${{\mathscr{P}}}_{{\rm{c}}}=1\,,{{\mathscr{P}}}_{{\rm{i}}}=0$ (a single back-scattering impurity in an ideal conductor) and *n* ^**T**^ = (0, 0, …, 1_*i*_, … 0) (single-particle scattering in the *i*-th channel) into Eq. (2) leads to the scaling dimensions Eq. (8).

## Summary

We have developed a universal approach to the calculation of scaling dimensions of generic scattering operators in multi-channel Luttinger liquids in arbitrary configuration, i.e when some of channels are ideal (conducting) and others are interrupted (insulating). The problem was reduced to a single matrix equation. The approach allowed to analyse a finite array of interacting Luttinger liquids and define the set of parameters where insulating bulk coexists with conducting edges. The situation is similar to the one observed in the quantum Hall systems or topological insulators. The model under consideration required neither magnetic field nor spin-orbit interaction to observe a quantised low-temperature edge transport.

## Electronic supplementary material

Supplementary Information
