# Supplementary material for: Interaction-induced edge states in anisotropic non-Fermi liquids
Source: Sci Rep. 2017 Jun 14;7:3550. doi: 10.1038/s41598-017-03823-5 (PMC5471236; doi:10.1038/s41598-017-03823-5)
Supplement: Supplementary file 1 — Supplementary Information [file 41598_2017_3823_MOESM1_ESM.pdf]

SUPPLEMENTARY INFORMATION FOR "INTERACTION-INDUCED EDGE STATES IN ANISOTROPIC NON-FERMI LIQUIDS"

I. V. Yurkevich

A. The Lagrangian

Any single one-dimensional system of fermions or bosons in the low-energy regime is described by the Hamiltonian[1]:

$$\hat{H}_1 = \frac{v}{8\pi}\int \mathrm{d}x \left[\frac{1}{K}\left(\partial_x\hat{\theta}\right)^2 + K\left(\partial_x\phi\right)^2\right]. \tag{A.1}$$

The velocity of low-lying excitations, v, and the Luttinger parameter, K, are related to the microscopic parameters of the system. The Luttinger parameter reflects interaction and statistics of the underlying particles: fermions with repulsion correspond to $K<1$ and fermions with attraction (or bosons with repulsion) are described by the universal Hamiltonian Eq. (A.1) with $K>1$. The density of excitations, $\hat{\rho}$, and current, $\hat{j}$, operators are related to the fields $\hat{\theta}$ and $\hat{\phi}$ as follows:

$$\hat{\rho} = \frac{1}{2\pi}\partial_x\hat{\theta}\,, \quad \hat{j} = \frac{1}{2\pi}\partial_x\hat{\phi}\,. \tag{A.2}$$

Taking into that $\hat{\theta}$- and $\hat{\phi}$-fields are mutually conjugate[1],

$$\left[\hat{\theta}\,,\partial_x\hat{\phi}\right] = 4\pi i\,\delta(x-x')\,, \tag{A.3}$$

one can switch to the field-theoretic description using the Lagrangian

$$\mathcal{L}_1 = \frac{1}{8\pi}\left[2\partial_t\,\phi\partial_x\theta - \frac{v}{K}\left(\partial_x\theta\right)^2 - vK\left(\partial_x\phi\right)^2\right]. \tag{A.4}$$

A set of one-dimensional wires, each described by its own velocity and the Luttinger parameter, without inter-wire interactions is governed by the Lagrangian

$$\mathcal{L}_N = \frac{1}{8\pi}\sum_i^N\left[2\partial_t\,\phi_i\partial_x\theta_i - \frac{v_i}{K_i}\left(\partial_x\theta\right)^2 - v_iK_i\left(\partial_x\phi\right)^2\right]. \tag{A.5}$$

Allowing density-density and current-current inter-wire interactions, we come to a generalised quadratic form

$$\mathcal{L} = \frac{1}{8\pi}\sum_{i,j=1}^N\left[2\delta_{ij}\partial_t\,\phi_i\partial_x\theta_i - V_+^{ij}\left(\partial_x\theta\right)^2 - V_-^{ij}\left(\partial_x\phi\right)^2\right]. \tag{A.6}$$

where

$$V_+^{ij} = \delta_{ij}\frac{v_i}{K_i} + u_+^{ij}\,, \quad V_-^{ij} = \delta_{ij}\,v_iK_i + u_-^{ij}\,. \tag{A.7}$$

Here matrix elements $u_\pm^{ij}$ describe interaction strength between wires i and j. The absence of the cross-terms between current and density is justified by the assumed inversion symmetry under which $\rho_i = \partial_x\theta_i/2\pi \to \rho_i$ and $j_i = \partial_x\phi/2\pi \to -j_i$. The Lagrangian Eq. (A.6) contains only smooth parts of the densities corresponding to forward particle-particle scattering. We neglected oscillating contributions (so-called g_1-processes[1]) assuming particle-particle interaction radius much lager than the mean inter-particle distance.

The Lagrangian Eq. (A.6) acquires a compact form after the introduction of the vectors-fields $\boldsymbol{\theta} = (\theta_1\,,\dots,\theta_N)$ and $\boldsymbol{\phi} = (\phi_1\,,\dots,\phi_N)$:

$$\mathcal{L} = \frac{1}{8\pi}\left[2\partial_t\,\boldsymbol{\phi}\partial_x\boldsymbol{\theta} - \partial_x\boldsymbol{\theta}^{\mathrm{T}}\,\hat{V}_+\,\partial_x\boldsymbol{\theta} - \partial_x\boldsymbol{\phi}^{\mathrm{T}}\,\hat{V}_-\,\partial_x\boldsymbol{\phi}\right]. \tag{A.8}$$

Even more compact form is achieved by the introduction of the composite vector $\boldsymbol{\Psi}^{\mathrm{T}} = (\boldsymbol{\theta}^{\mathrm{T}}\,,\boldsymbol{\phi}^{\mathrm{T}})$ and integration by parts:

$$\mathcal{L}_0 = \frac{1}{8\pi}\boldsymbol{\Psi}^{\mathrm{T}}\left[\hat{\tau}_1\,\partial_t + \hat{V}\,\partial_x\right]\partial_x\boldsymbol{\Psi}\,, \qquad \hat{V} = \mathrm{diag}[\hat{V}_+\,,\hat{V}_-]\,. \tag{A.9}$$

Here $\hat{\tau}_1$ is the Pauli matrix in (θ,ϕ)-space. The Eq. (A.9) describes a generic multi-channel (multi-wire) without inter-channel hybridization and it is used as a starting point in the main text.

B. Lagrangian diagonalisation for translation invariant problem

The density-density, $\hat{V}_+$, and current-current, $\hat{V}_-$, interaction matrices are real and positive-definite. The latter is the requirement for the the energy to be bounded from below. the Lagrangian two real (all matrices in this Appendix will be real) symmetric positive definite matrices (the positiveness is required for stability)

$$\hat{V}_\pm = \hat{V}_\pm^{\mathrm{T}}\,, \quad \hat{V}_\pm \succ 0\,, \tag{B.1}$$

we have uniquely defined square roots because positive definite matrices have positive eigenvalues:

$$\hat{V} = \hat{V}_\pm^{\mathrm{T}}\,, \quad \hat{V} \succ 0\,, \tag{B.2}$$

$$\hat{V} = \hat{O}\,\hat{\lambda}\,\hat{O}^{\mathrm{T}}\,, \quad \hat{O}\,\hat{O}^{\mathrm{T}} = 1 \quad \lambda_i \geq 0\,, \tag{B.3}$$

$$\hat{V}^{1/2} = \hat{O}\,\hat{\lambda}^{1/2}\,\hat{O}^{\mathrm{T}}\,. \tag{B.4}$$

The product (which is a non-symmetric matrix) of any two positive definite matrices has also positive eigenvalues:

$$\hat{V}_-\,\hat{V}_+ = \hat{V}_-^{1/2}\left[\hat{V}_-^{1/2}\hat{V}_+\,\hat{V}_-^{1/2}\right]\hat{V}_-^{-1/2} \sim \hat{V}_-^{1/2}\hat{V}_+\,\hat{V}_-^{1/2} \succ 0\,. \tag{B.5}$$

Since the product is similar to a positive definite matrix (the last one in the equation above), they have identical eigenvalues, i.e. positive ones. Lets us call them v_i^2 and the diagonal matrix of eigenvalues $\hat{v}^2 = \mathrm{diag}(v_1^2, v_2^2, ..., v_N^2)$. Then we can diagonalise this product by similarity transformation with some matrix $\hat{M}$:

$$\hat{V}_-\,\hat{V}_+ = \hat{M}\,\hat{v}^2\,\hat{M}^{-1}\,. \tag{B.6}$$

It turns out that one can solve this equation for $\hat{V}_+$ and $\hat{V}_-$. To do it, let us write equation transpose to Eq. (B.6)

$$\hat{V}_+\,\hat{V}_- = \hat{M}^{-\mathrm{T}}\,\hat{v}^2\,\hat{M}^{\mathrm{T}}\,, \tag{B.7}$$

then find $\hat{V}_+$ from the last equation and plug it back into Eq. (B.6). The resultant equation will have the following form

$$\hat{\Lambda}_-\,\hat{v}^2 = \hat{v}^2\,\hat{\Lambda}_-\,, \quad \hat{\Lambda}_- = \hat{M}^{-\mathrm{T}}\,\hat{V}_-\,\hat{M}^{-1}\,. \tag{B.8}$$

The only solution of this equation is Λ_- which is a diagonal matrix. Repeating the same trick with $\hat{V}_+$ we find similar expression:

$$\hat{\Lambda}_+\,\hat{v}^2 = \hat{v}^2\,\hat{\Lambda}_+\,, \quad \hat{\Lambda}_+ = \hat{M}\,\hat{V}_+\,\hat{M}^{\mathrm{T}}\,. \tag{B.9}$$

and similar conclusion: Λ_- is a diagonal matrix. As the result, we can claim that

$$\hat{V}_- = \hat{M}\,\hat{\Lambda}_-\,\hat{M}^{\mathrm{T}}\,, \tag{B.10}$$

$$\hat{V}_+ = \hat{M}^{-\mathrm{T}}\,\hat{\Lambda}_+\,\hat{M}^{-1}\,. \tag{B.11}$$

To provide consistency with the Eq. (B.6), we have to demand

$$\hat{\Lambda}_-\,\hat{\Lambda}_+ = \hat{v}^2\,. \tag{B.12}$$

There is a freedom here, but we would like to have both Λ's equal ($\hat{\Lambda}_- = \hat{\Lambda}_+ = \hat{v}$) because then they are interpreted as velocities in the new channels. Our final result:

$$\hat{V}_- = \hat{M}\,\hat{v}\,\hat{M}^{\mathrm{T}}\,, \tag{B.13}$$

$$\hat{V}_+ = \hat{M}^{-\mathrm{T}}\,\hat{v}\,\hat{M}^{-1}\,. \tag{B.14}$$

Please note that velocities are not eigenvalues of interaction matrices, their squares are eigenvalues of the products of interaction matrices. The important consequence of these two equations is that if they are written in the form

$$\hat{v} = \hat{M}^{-1}\,\hat{V}_-\,\hat{M}^{-\mathrm{T}} = \hat{M}^{\mathrm{T}}\,\hat{V}_+\,\hat{M}\ , \tag{B.15}$$

then from the second equality one can get relation between two arbitrary symmetric positive-definite matrices

$$\hat{K}\,\hat{V}_+\,\hat{K} = \hat{V}_-\,, \tag{B.16}$$

that may be treated as the equation for the Luttinger matrix $\hat{K}$ which was defined as

$$\hat{K} = \hat{M}\,\hat{M}^{\mathrm{T}}\,, \tag{B.17}$$

and, therefore, is real symmetric positive definite $N \times N$ matrix. This completes the proof of our main Eq. (1).

C. Translation invariant sLL

First of all, one has to find the retarded Green function (we will suppress the superscript for 'retarded' component in what follows) corresponding to the ideal (translation invariant) multi-channel Luttinger liquid model (Lagrangian Eq. (4) in the main text) using transformation Eq. B.13 from the next Section II:

$$\hat{G}_0(x;\omega) = 2\int \mathrm{d}q \begin{pmatrix} -\hat{V}_+ q^2 & \omega q \\ \omega q & -\hat{V}_- q^2 \end{pmatrix}^{-1} e^{iqx} = \hat{\mathcal{M}}\, \hat{g}_0(x;\omega)\, \hat{\mathcal{M}}^{\mathrm{T}} \,. \tag{C.1}$$

Here $\hat{g}_0$ is diagonal in channel indices Green function that describes set of N uncorrelated one-dimensional Luttinger liquids with renormalised (by interactions) velocities:

$$\hat{g}_0(x;\omega) = 2\int \mathrm{d}q \begin{pmatrix} -\hat{v}q^2 & \omega q \\ \omega q & -\hat{v}q^2 \end{pmatrix}^{-1} e^{iqx} = -\frac{2\pi i}{\omega} \begin{pmatrix} \hat{1} & \mathrm{sgn}(x) \\ \mathrm{sgn}(x) & \hat{1} \end{pmatrix} e^{i\hat{\omega}|x|} \,.$$

The transformation is performed in density and current sectors without mixing them:

$$\hat{\mathcal{M}} = \begin{pmatrix} \hat{M} & 0 \\ 0 & \hat{M}^{-\mathrm{T}} \end{pmatrix} \,. \tag{C.2}$$

We have also introduced the notations $\hat{\omega} = \frac{\omega}{\hat{v}}$ for the channel dependent frequency renormalisation. The ideal multi-channel LL is described by the following Green function

$$G_0(x;\omega) = -\frac{2\pi i}{\omega} \begin{pmatrix} \hat{K} & \mathrm{sgn}(x) \\ \mathrm{sgn}(x) & \hat{K}^{-1} \end{pmatrix} e^{i\hat{\omega}|x|} \,, \quad \hat{K} = \hat{M}\,\hat{M}^{\mathrm{T}} \,.$$

where we introduced the Luttinger $\hat{K}$-matrix derived in the B and introduced in the main text Eq. (1).

If we needed equal coordinate correlation functions only (let us say at $x=0$), we could work out the corresponding effective Lagrangian. At zero-temperature we would have to invert the causal local Green function ($G^{\mathrm{c}} = -2\pi\, i/|\omega|$) to come to the result:

$$\mathcal{L}_{\mathrm{ideal}} = i\int \frac{\mathrm{d}\omega}{2\pi}\, |\omega| \left[\boldsymbol{\theta}_\omega^\dagger\, \hat{K}^{-1}\, \boldsymbol{\theta}_\omega + \boldsymbol{\phi}_\omega^\dagger\, \hat{K}\, \boldsymbol{\phi}_\omega \right] \tag{C.3}$$

D. Local impurity

Perturbing translation invariant system by a local impurity, one may find that the system is unstable in the sense that the scaling dimension of this perturbation is smaller than one (which is the physical dimension of a local term in $1+1$ dimensional system). This means that a perturbation theory would be divergent and allegedly translation invariant configuration is a bad initial approximation. In terms of transport, this fact suggests that some (all or few) of the channels (wires) become insulating and not ideally conducting as it is assumed in a translation invariant configuration. To test stability of such an inhomogeneous configuration where some channels are conducting while others are insulating we have to introduce two subspaces of conducting, **C**, and insulating, **I**, channels. Since we have no *á priori* knowledge which configuration will be stable (and, therefore, may be called a phase) against all meaningful perturbations, we must devise generic approach allowing treatment of all possible configurations.

The translation invariant channels, $i \subset \mathbf{C}$, are described by continuous θ_i - and ϕ_i -fields. The channels that, as an initial guess, are assumed to be insulating, $i \subset \mathbf{I}$, should be described by continuous θ_i-fields which vanish at the position of interruption (at the origin $x=0$). The boundary conditions for insulating (disjoint at $x=0$) channel follow from the requirement that current (written as $j_i(x,t) = -\partial_t \theta_i(x,t)/2\pi$) at the tip of an insulating channel vanishes. Instead of using the boundary conditions described above, we will do a trick by adding to the translation invariant Lagrangian, $\mathcal{L}_0$, an auxiliary term

$$\mathcal{L}_\xi = \mathcal{L}_0 - \frac{1}{2}\boldsymbol{\theta}^{\mathrm{T}}\, \hat{\zeta}\, \boldsymbol{\theta} \,, \quad \hat{\zeta} = \mathrm{diag}(\zeta_1\,, ..., \zeta_N) \,. \tag{D.1}$$

The correlations found from the auxiliary Lagrangian $\mathcal{L}_\xi$ become true correlations of an inhomogeneous configuration after the limit

$$\zeta_i \to \begin{cases} 0\,, & i \subset \mathbf{C}\,, \\ \infty\,, & i \subset \mathbf{I}\,. \end{cases} \tag{D.2}$$

The trick allows dealing with all configurations indifferently and the limit Eq. (D.2), effectively suppressing currents in insulating channels (subset **I**) and leaving unaffected conducting channels (subset **C**), should be taken at the very end of calculations. In what follows, we will refer to this limiting procedure simply as ξ-limit (the name will become clear later when ζ will be renormalised and renamed to ξ). The auxiliary Lagrangian involves the auxiliary Green function

$$\mathcal{L}_\xi = \frac{1}{2}\mathbf{\Psi}^{\mathrm{T}}\,\hat{G}_\xi^{-1}\,\mathbf{\Psi}\,,\quad \hat{G}_\xi^{-1} = \hat{G}_0^{-1} - \hat{\Sigma}\,,\quad \hat{\Sigma}(x,x') = \begin{pmatrix} \hat{\zeta} & 0 \\ 0 & 0 \end{pmatrix} \delta(x)\delta(x')\,. \tag{D.3}$$

To calculate scaling dimensions of scattering operators one needs correlations

$$\langle \mathbf{\Psi}(x,t) \otimes \mathbf{\Psi}^{\mathrm{T}}(x',t')\rangle = iG_\xi(x,x';t,t')\,. \tag{D.4}$$

The Green function is defined on real time axis for zero temperature or on the Keldysh contour for a finite temperature. The Green function can be found with the use of scattering $\hat{T}$-matrix. The equation for retarded Green function in $(\omega\,,x)$-representation is given by

$$\hat{G}_\xi^{\mathrm{R}}(x,x';\omega) = \hat{G}_0^{\mathrm{R}}(x-x';\omega) + \hat{G}_0^{\mathrm{R}}(x;\omega)\,\mathrm{T}_\xi^{\mathrm{R}}(\omega)\,\hat{G}_0^{\mathrm{R}}(-x';\omega)\,, \tag{D.5}$$

where $\hat{T}$-matrix can be expressed through the (local) self-energy $\hat{\Sigma}$, generated by the boundary term

$$\hat{T}_\xi^{\mathrm{R}}(\omega) = \hat{\Sigma}^{\mathrm{R}}\left[1 - \hat{G}_0^{\mathrm{R}}(0;\omega)\hat{\Sigma}^{\mathrm{R}}\right]^{-1}\,,\quad \hat{\Sigma}^{\mathrm{R}} = \begin{pmatrix} \hat{\zeta} & 0 \\ 0 & 0 \end{pmatrix}\,. \tag{D.6}$$

Now we can calculate the $\hat{T}$-matrix

$$\hat{T}_\xi = \frac{\omega}{2\pi i}\begin{pmatrix} \hat{R}_\xi & 0 \\ 0 & 0 \end{pmatrix}\,,\quad \hat{R}_\xi = \frac{1}{\hat{K} + \hat{\xi}^{-1}}\,,\quad \hat{\xi} = \frac{2\pi i}{\omega}\,\hat{\zeta}\,, \tag{D.7}$$

introducing dimensionless auxiliary diagonal matrix $\hat{\xi}$ which justifies previously used notation for ξ-limit. The Green function (before the ξ-limit) can be written as

$$\hat{G}_\xi = -\frac{2\pi i}{\omega}\left[\begin{pmatrix} \hat{K} & s_{x-x'} \\ \mathrm{s}_{x-x'} & \hat{K}^{-1} \end{pmatrix} e^{i\hat{\omega}|x-x'|} - \begin{pmatrix} \hat{K}\,\hat{R}_\xi\,\hat{K} & \hat{K}\,\hat{R}_\xi\,s_{-x'} \\ \mathrm{s}_x\,\hat{R}_\xi\,\hat{K} & \mathrm{s}_x\,\hat{R}_\xi\,s_{-x'} \end{pmatrix} e^{i\hat{\omega}(|x|+|x'|)}\right]\,, \tag{D.8}$$

where the notation $\mathrm{s}_x = \mathrm{sgn}(x)$ has been used. Since calculation of scaling dimension of a local perturbation requires only asymptotes of the correlations between fields at the origin ($x = \pm 0, x' = \pm 0$) we may use the 'local' limit of Eq. (D.8):

$$\hat{G}_\xi(x,x';\omega)_{x,x'\to 0} \to -\frac{2\pi i}{\omega}\begin{pmatrix} (1-\hat{K}\hat{R}_\xi)\hat{K} & s_{x-x'} + \hat{K}\,\hat{R}_\xi\,s_{x'} \\ \mathrm{s}_{x-x'} - \mathrm{s}_x\,\hat{R}_\xi\,\hat{K} & \hat{K}^{-1} + \hat{R}_\xi\,s_{xx'} \end{pmatrix}\,. \tag{D.9}$$

E. Scaling dimension of the scattering term

The most general term describing process of simultaneous backscattering of few particles in the conducting channels and accompanying it transfer (tunneling) of particles across the cut in the insulating channels is written as

$$\mathcal{L}_{\mathrm{pert}} = \sum_{\mathbf{n}} \mathcal{L}_{\mathbf{n}}\,,\quad \mathcal{L}_{\mathbf{n}} = v_{\mathbf{n}}\,e^{i\mathbf{n}^{\mathrm{T}}\mathbf{\Phi}} + \mathrm{c.c.}\,,\quad \mathbf{\Phi} = \boldsymbol{\theta}_{\mathrm{c}} + \boldsymbol{\varphi}_{\mathrm{i}}\,, \tag{E.1}$$

where we defined integer-valued vector $\mathbf{n}^{\mathrm{T}} = (n_1, n_2,\,\dots, n_N)$ and a new field $\boldsymbol{\varphi}$ describing the old ϕ-fields discontinuity around the scatterer (placed at the origin):

$$\boldsymbol{\varphi}_{\mathrm{i}} = \frac{1}{2}\left[\boldsymbol{\phi}_{\mathrm{i}}(x=+0) - \boldsymbol{\phi}_{\mathrm{i}}(x=-0)\right]\,. \tag{E.2}$$

There are two different processes that appear in the scattering term Eq. (E.1). First, it is backscattering in conducting channels where only $\boldsymbol{\theta}$-fields from conducting channels, $\boldsymbol{\theta}_{\mathrm{c}}$, define this process. Second, the tunneling in insulating

channels occurs and it is governed by the fields discontinuity Eq. (E.2). We may introduce the projection operators that act in the channel space and separate conducting channels from insulating ones:

$$\boldsymbol{\theta}_{\rm c} = \hat{\mathcal{P}}_{\rm c}\, \boldsymbol{\theta}\,, \quad \boldsymbol{\varphi}_{\rm i} = \hat{\mathcal{P}}_{\rm i}\, \boldsymbol{\varphi}\,, \quad \boldsymbol{\Phi} = \mathcal{P}_{\rm c}\, \boldsymbol{\theta} + \mathcal{P}_{\rm i}\, \boldsymbol{\varphi} = \boldsymbol{\theta}_{\rm c} + \boldsymbol{\varphi}_{\rm i}\,. \tag{E.3}$$

The Eq. (E.1) represents the most general perturbation applied to an arbitrary configuration if we neglect (as it was discussed in the introduction) scattering accompanied by a change of the channel index. This is justified when conservation laws prohibit change of the quantum number corresponding to the nomenclature of channels or when overlap between wavefunctions belonging to different wires (channels) is small. But we would like to stress that we restrict ourselves to a perturbation given above only to keep it simple. If one needs to account for a specific scattering process, the only thing which has to be done is relabeling of *chiral* channels to make sure that the important scattering event occurs inside the same non-chiral channel. Such a procedure will corrupt the matrices $\hat{V}_{\pm}$ but otherwise the whole approach will be intact.

Not all correlations present in Eq. (D.9) are needed for calculation of scaling dimensions of the scattering terms. All the necessary for this purpose correlations are contained in the reduced Green function:

$$i\,\mathcal{G}_{\xi} = \begin{pmatrix} \langle \boldsymbol{\theta}_{\rm c} \otimes \boldsymbol{\theta}_{\rm c}^{\rm T} \rangle & \langle \boldsymbol{\theta}_{\rm c} \otimes \boldsymbol{\varphi}_{\rm i}^{\rm T} \rangle \\ \langle \boldsymbol{\varphi}_{\rm i} \otimes \boldsymbol{\theta}_{\rm c}^{\rm T} \rangle & \langle \boldsymbol{\varphi}_{\rm i} \otimes \boldsymbol{\varphi}_{\rm i}^{\rm T} \rangle \end{pmatrix}. \tag{E.4}$$

The retarded reduced Green function is readily extracted from Eq. (D.9)

$$i\,\mathcal{G}_{\xi} = \frac{2\pi}{\omega} \begin{pmatrix} \hat{\mathcal{P}}_{\rm c} \left[\hat{K}^{-1} + \hat{\xi}\right]^{-1} \hat{\mathcal{P}}_{\rm c} & \hat{q}_{\xi} \\ -\hat{q}_{\xi}^{\rm T} & \hat{\mathcal{P}}_{\rm i} \left[\hat{K} + \hat{\xi}^{-1}\right]^{-1} \hat{\mathcal{P}}_{\rm i}\,, \end{pmatrix}, \tag{E.5}$$

where the off-diagonal elements are given by the following expression:

$$\hat{q}_{\xi} = \hat{\mathcal{P}}_{\rm c}\, \hat{K} \left[\hat{K} + \hat{\xi}^{-1}\right]^{-1} \hat{\mathcal{P}}_{\rm i}\,. \tag{E.6}$$

And finally, we have to perform the ξ-limit. It is convenient to do it after presenting $\hat{K}$-matrix in the block form distinguishing conducting and insulating subspaces of channels:

$$\hat{K} = \begin{pmatrix} \hat{K}_{\rm cc} & \hat{K}_{\rm ci} \\ \hat{K}_{\rm ic} & \hat{K}_{\rm ii} \end{pmatrix}, \quad \hat{K}^{-1} = \begin{pmatrix} \hat{\hat{K}}_{\rm cc} & \hat{\hat{K}}_{\rm ci} \\ \hat{\hat{K}}_{\rm ic} & \hat{\hat{K}}_{\rm ii} \end{pmatrix}, \tag{E.7}$$

with blocks defined by the projectors

$$\hat{K}_{\mu\nu} = \hat{\mathcal{P}}_{\mu}\, \hat{K}\, \hat{\mathcal{P}}_{\nu}\,, \quad \hat{\hat{K}}_{\mu\nu} = \hat{\mathcal{P}}_{\mu}\, \hat{K}^{-1}\, \hat{\mathcal{P}}_{\nu}\,, \quad \mu,\nu = {\rm c},{\rm i}\,.$$

The ξ-limit brings the final result for the correlations needed to find scaling dimensions of the perturbations Eq. (E.1) into the form:

$$\lim_{\xi} \mathcal{G}_{\xi} \equiv \mathcal{G} = -\frac{2\pi\, i}{\omega} \begin{pmatrix} \hat{\hat{K}}_{\rm cc}^{-1} & \hat{q} \\ -\hat{q}^{\rm T} & \hat{K}_{\rm ii}^{-1} \end{pmatrix}, \quad \hat{q} = \hat{K}_{\rm ci}\, \hat{K}_{\rm ii}^{-1}\,. \tag{E.8}$$

Using results from the previous section, we can write correlations in the time domain (these are actually greater or lesser Green functions with the corresponding infinitesimal imaginary shift which is not written explicitly below):

$$\begin{aligned} \langle \boldsymbol{\theta}_{\rm c}(t) \otimes \boldsymbol{\theta}_{\rm c}^{\rm T}(t') \rangle &= -2\hat{\hat{K}}_{\rm cc}^{-1}\, \ln(t-t')\,, \\ \langle \boldsymbol{\varphi}_{\rm i}(t) \otimes \boldsymbol{\varphi}_{\rm i}^{\rm T}(t') \rangle &= -2\hat{K}_{\rm ii}^{-1}\, \ln(t-t')\,. \end{aligned} \tag{E.9}$$

There are also cross-correlations (unimportant for our purposes as it will be seen later):

$$\begin{aligned} \langle \boldsymbol{\theta}_{\rm c}(t) \otimes \boldsymbol{\varphi}_{\rm i}^{\rm T}(t') \rangle &= -2\hat{q}\, \ln(t-t')\,, \\ \langle \boldsymbol{\varphi}_{\rm i}(t) \otimes \boldsymbol{\theta}_{\rm c}^{\rm T}(t') \rangle &= 2\hat{q}^{\rm T}\, \ln(t-t')\,. \end{aligned} \tag{E.10}$$

The scaling dimensions, $\Delta[\mathbf{n}]$, of exponentials in Eq. (E.1) are defined by the correlations:

$$\langle e^{i\mathbf{n}^{\rm T}\boldsymbol{\Phi}(t)}\, e^{-i\mathbf{n}^{\rm T}\boldsymbol{\Phi}(t')} \rangle \sim \exp\left[\mathbf{n}^{\rm T} \langle \boldsymbol{\Phi}(t) \otimes \boldsymbol{\Phi}^{\rm T}(t') \rangle \mathbf{n}\right] = (t-t')^{-2\Delta[\mathbf{n}]}\,. \tag{E.11}$$

Since cross-correlations in Eq. (E.10) are transposed to each other with the minus sign, they do not contribute to the correlation function defining scaling dimension in Eq. (E.11). Using Eqs. (E.9), one can write down scaling dimensions in the following form:

$$\dim[\mathcal{L}_{\mathbf{n}}] = \Delta[\mathbf{n}] = \mathbf{n}_{\rm c}^{\rm T}\,\hat{\hat{\mathbf{K}}}_{\rm cc}^{\mathbf{-1}}\,\mathbf{n}_{\rm c} + \mathbf{n}_{\rm i}^{\rm T}\hat{\mathbf{K}}_{\rm ii}^{\mathbf{-1}}\,\mathbf{n}_{\rm i}\,, \quad \mathbf{n}_{\rm c} = \mathcal{P}_{\rm c}\,\mathbf{n}\,, \quad \mathbf{n}_{\rm i} = \mathcal{P}_{\rm i}\,\mathbf{n}\,. \tag{E.12}$$

Finally, using properties of the projectors the scaling dimension can finally be written in the form presented earlier in the Introduction, Eq. (2):

$$\Delta[\mathbf{n}] = \mathbf{n}^{\rm T}\,\hat{\Delta}\,\mathbf{n}\,, \quad \hat{\Delta}^{-1} = \mathcal{P}_{\rm c}\,\hat{K}^{-1}\,\mathcal{P}_{\rm c} + \mathcal{P}_{\rm i}\,\hat{K}\,\mathcal{P}_{\rm i}\,. \tag{E.13}$$

F. Duality

Let us compare two arbitrary configurations different by only one element, i.e. some channel is assumed to be conducting in the first configuration and insulating in another one. We will call it first channel because we have not arranged channels spatially and the enumeration is irrelevant so far. According to the Eq. (**??**), one-particle backscattering in the first channel is governed by the scaling dimension

$$\Delta_{\rm c} = \left[\left(\hat{\hat{\mathcal{P}}}_{\rm c}\,\hat{K}^{-1}\,\hat{\hat{\mathcal{P}}}_{\rm c}\right)^{-1}\right]_{11}\,, \tag{F.1}$$

where the inversion is taken in the subspace of conducting channels. If the same wire was insulating, in the same environment of other channels, it would be disturbed by a tunneling with the scaling dimension given by

$$\Delta_{\rm i} = \left[\left(\hat{\hat{\mathcal{P}}}_{\rm i}\,\hat{K}\,\hat{\hat{\mathcal{P}}}_{\rm i}\right)^{-1}\right]_{11}\,. \tag{F.2}$$

Since in both cases the 'environment' is the same, we can introduce projection operators acting in $(N-1)\times(N-1)$ space of all channels except the first one:

$$\hat{\hat{\mathcal{P}}}_{\rm c} = \begin{pmatrix} 1 & 0 \\ 0 & \hat{P}_{\rm c} \end{pmatrix}\,, \quad \hat{\hat{\mathcal{P}}}_{\rm c} = \begin{pmatrix} 1 & 0 \\ 0 & \hat{P}_{\rm i} \end{pmatrix}\,, \quad \hat{P}_{\rm c} + \hat{P}_{\rm i} = 1\,. \tag{F.3}$$

It is instructive to use $\hat{K}$-matrix representation in the block form separating conducting and insulating channels (see Eq. (E.7)):

$$\hat{K} = \begin{pmatrix} \hat{K}_{\rm cc} & \hat{K}_{\rm ci} \\ \hat{K}_{\rm ic} & \hat{K}_{\rm ii} \end{pmatrix}\,. \tag{F.4}$$

The channel one belongs to the **C**-subspace in the one configuration and to the **I**-subspace in the other. Since the projection of the inverse matrix can be calculated as

$$\hat{\mathcal{P}}_{\rm c}\,\hat{K}^{-1}\,\hat{\mathcal{P}}_{\rm c} = \left(\hat{K}_{\rm cc} - \hat{K}_{\rm ci}\hat{K}_{\rm ii}\hat{K}_{\rm ic}\right)^{-1}\,, \tag{F.5}$$

we get the expression for the scaling dimension of the operator perturbing the first channel when it is in a conducting state:

$$\Delta_{\rm c} = \left[\hat{K}_{\rm cc} - \hat{K}_{\rm ci}\hat{K}_{\rm ii}^{-1}\hat{K}_{\rm ic}\right]_{11} = K_{11} - \mathbf{k}_{\rm i}^{\rm T}\,\hat{K}_{\rm ii}^{-1}\,\mathbf{k}_{\rm i}\,, \tag{F.6}$$

where we have to define an auxiliary $\mathbf{k}_i$-vector

$$\mathbf{k}_{\rm i} = \hat{P}_{\rm i}\,\mathbf{k}\,, \quad k^{\rm T} = (K_{12}\,, K_{13}\,, \ldots\,, K_{1N})\,. \tag{F.7}$$

Calculating alternative scaling dimension we need another projected matrix

$$\hat{\hat{\mathcal{P}}}_{\rm i}\,\hat{K}\,\hat{\hat{\mathcal{P}}}_{\rm i} = \begin{pmatrix} K_{11} & \mathbf{k}_i^{\rm T} \\ \mathbf{k}_i & \hat{K}_{\rm ii} \end{pmatrix}\,. \tag{F.8}$$

To find the inverse we may use cofactor matrix with $(1,1)$-minor given by $\det\hat{K}_{\rm ii}$:

$$\Delta_{\rm i} = \left[\begin{pmatrix} K_{11} & \mathbf{k}_i^{\rm T} \\ \mathbf{k}_i & \hat{K}_{\rm ii} \end{pmatrix}^{-1} \right]_{11} = \frac{\det \hat{K}_{\rm ii}}{\det(\hat{\hat{\mathcal{P}}}_{\rm i}\, \hat{K}\, \hat{\hat{\mathcal{P}}}_{\rm i})} \, . \tag{F.9}$$

Then we can the following reduction from $N \times N$ to $(N-1) \times (N-1)$ matrix representation:

$$\det \left(\hat{\hat{\mathcal{P}}}_{\rm i}\, \hat{K}\, \hat{\hat{\mathcal{P}}}_{\rm i} \right) = \det\hat{K}_{\rm ii} \det \left[K_{11} - \mathbf{k}_i^{\rm T}\, \hat{K}_{\rm ii}^{-1}\, \mathbf{k}_i \right] \, . \tag{F.10}$$

Now one can see that the scaling dimension of the perturbation above insulating state of the first channel is equal to

$$\Delta_{\rm i} = \left[K_{11} - \mathbf{k}_i^{\rm T}\, \hat{K}_{\rm ii}^{-1}\, \mathbf{k}_i \right]^{-1} \, . \tag{F.11}$$

The comparison of the results Eq. (F.6) and Eq. (F.11) proves the duality between scaling dimensions of perturbations in a single channel when it switches from conducting to insulating state:

$$\Delta_{\rm c}\, \Delta_{\rm i} = 1 \, . \tag{F.12}$$

The duality equation is used in the text to prove that if all inner (bulk) wires are not switching their state, the edge wire can be in one of two states that are mutually exclusive, i.e. conducting and insulating phases of the edge exist in different parametric regions and never mix up, the unstable fixed point is impossible due to Eq. (F.12) and phases are robust against arbitrary strength of impurity scattering.

[1] T. Giamarchi. "Quantum physics in one dimension". Caledon Press, Oxford (2003).
